# Cellular glycosylation affects Herceptin binding and sensitivity of breast cancer cells to doxorubicin and growth factors

**DOI:** 10.1038/srep43006

**Published:** 2017-02-22

**Authors:** Diluka Peiris, Alexander F. Spector, Hannah Lomax-Browne, Tayebeh Azimi, Bala Ramesh, Marilena Loizidou, Hazel Welch, Miriam V. Dwek

**Affiliations:** 1 Attana AB, Bjornnasvagen 21, SE-11419, Stockholm, Sweden; 2 Division of Surgery and Interventional Science, UCL Medical School Royal Free Campus, Rowland Hill Street, London, NW3 2PF, UK; 3 Department of Biomedical Sciences, Faculty of Science and Technology, University of Westminster, 115 New Cavendish St, W1W 6UW, UK

## Abstract

Alterations in protein glycosylation are a key feature of oncogenesis and have been shown to affect cancer cell behaviour perturbing cell adhesion, favouring cell migration and metastasis. This study investigated the effect of N-linked glycosylation on the binding of Herceptin to HER2 protein in breast cancer and on the sensitivity of cancer cells to the chemotherapeutic agent doxorubicin (DXR) and growth factors (EGF and IGF-1). The interaction between Herceptin and recombinant HER2 protein and cancer cell surfaces (on-rate/off-rate) was assessed using a quartz crystal microbalance biosensor revealing an increase in the accessibility of HER2 to Herceptin following deglycosylation of cell membrane proteins (deglycosylated cells B_max_: 6.83 Hz; glycosylated cells B_max_: 7.35 Hz). The sensitivity of cells to DXR and to growth factors was evaluated using an MTT assay. Maintenance of SKBR-3 cells in tunicamycin (an inhibitor of N-linked glycosylation) resulted in an increase in sensitivity to DXR (0.1 μM DXR P < 0.001) and a decrease in sensitivity to IGF-1 alone and to IGF-1 supplemented with EGF (P < 0.001). This report illustrates the importance of N-linked glycosylation in modulating the response of cancer cells to chemotherapeutic and biological treatments and highlights the potential of glycosylation inhibitors as future combination treatments for breast cancer.

Aberrant protein glycosylation is a well-established event in oncogenesis; shown to correlate with metastasis formation and resulting from changes in the expression levels and location of glycosyltransferases^1^,^2^,^3^. Our laboratory and others have identified cancer-associated glycoproteins that exhibit aberrant glycosylation^4^,^5^,^6^, such glycoproteins include mucins (for example MUC1^7^), integrins^8^ and cadherins^9^. Despite our understanding of the glycosylation changes occurring in cancer the effect on the binding of drugs to cell surface glycoproteins and on sensitivity of cancer cells to chemotherapeutic agents remains relatively poorly explored.

The human epidermal growth factor receptor 2 (HER2) protein, a member of the tyrosine kinase receptor family, is over-expressed in 25–30% of breast cancers, correlating with poor patient prognosis^10^. On binding to epidermal growth factor (EGF), HER2 can form homo- or heterodimers (with HER1, HER3 or HER4) triggering a series of events leading to the activation of members of the MAPK and PI3 kinase/AKT pathways. The overexpression of HER2 results in the constitutive activation of these pathways and cell proliferation. HER2 has been targeted with the monoclonal antibody drug Herceptin (Trastuzumab, Roche, Welwyn Garden City, UK) which upon binding induces a cytostatic effect associated with G1 arrest^11^. In a murine model, Herceptin was also shown to induce antibody dependent cell-mediated cytotoxity^12^.

Whilst the development of biological drugs such as Herceptin represent a breakthrough in the treatment of cancer, a significant number of HER2 positive patients either do not respond to, have innate, or develop acquired resistance to the treatment^13^. Herceptin resistance may arise - amongst others - as a result of epidermal growth factor (EGF) signalling through other HER receptors^14^, or signalling via the insulin-like growth factor receptor (IGFR)^15^,^16^ or through the activation of the PI3K/mTOR pathway^17^. A further proposed mechanism for Herceptin resistance is the physical blockade or masking of the HER2 receptor^18^, for example, the MUC4 molecule with its extended carbohydrate structure appears to serve as a barrier for biomolecular interactions in the extracellular environment^19^,^20^ and over-expression of MUC4 in the Herceptin resistant breast cancer cell line JIMT-1 has been shown to lead to diminished Herceptin binding and isolation of the receptor from its normal interaction and activation partner^21^.

The chemotherapeutic agent DXR, a member of the anthracycline family of antibiotics^22^ has been used in combination therapies and as a front-line treatment for lymphoma, ovarian cancer, lung and breast cancer^23^. Again, innate and acquired resistance to DXR, as well as to other chemotherapeutic agents, remains a major obstacle to the successful treatment of cancer^24^. As cell surface proteins are often heavily glycosylated it has been postulated that such glycosylation may affect epitope accessibility and drug binding to receptor proteins, similarly, the glycocalyx might affect the sensitivity of cancer cells to chemotherapeutic agents.

In this study it was hypothesised that N-linked glycosylation might impede the binding of Herceptin to HER2 in breast cancer and alter cancer cell sensitivity to DXR and growth factors. To study Herceptin-HER2 binding a cell based quartz crystal microbalance (QCM) system with adherent cancer cells grown on the surface of a biosensor chip was used. The cell-chip enabled an evaluation of the kinetics of interaction between Herceptin and HER2 in a quasi-physiological environment^25^,^26^,^27^. In contrast to conventional systems, where single receptors such as glycoprotein entities are investigated, the cell based QCM enables drug-receptor interactions to be studied in the presence of the other biomolecules present at the cell surface. The interaction between Herceptin and SK-OV-3 cells (a human ovarian carcinoma cell line) was recently investigated using this approach^28^ and we used the QCM-based system to study Herceptin binding to the HER2-over-expressing breast cancer cell line SKBR-3^29^ as well as to recombinant HER2 (rHER2) protein and to evaluate the effect of deglycosylation on Herceptin binding. In addition, breast cancer cells were grown in the presence of tunicamycin, an antibiotic that blocks the transfer of GlcNAc-1-P to dolichol-P, an essential early step in the intracellular production of N-linked glycans. The reduction in cell surface glycosylation following tunicamycin treatment was evaluated using the lectins wheat germ agglutinin (WGA) and Concanavilin A (Con A), carbohydrate binding proteins recognising N-linked glycans. The sensitivity of cancer cells to growth factors: EGF and IGF-1 alone and in combination, was evaluated following tunicamycin treatment and the role of glycosylation on sensitivity to DXR was determined. In summary, the current study evaluated the role of glycosylation on the binding of Herceptin and the sensitivity of breast cancer cells to DXR. In addition, the role of glycosylation on cellular sensitivity to growth factors (EGF, IGF-1) was evaluated.

## Results

**Figure 1:**
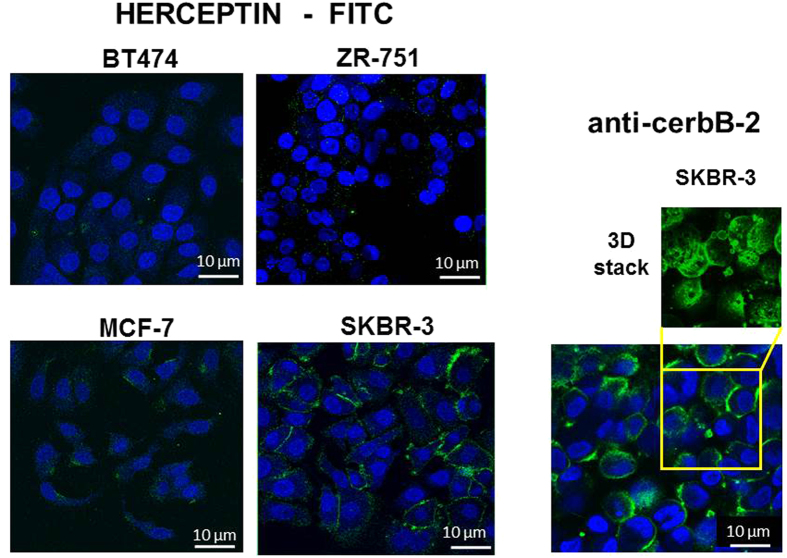
Herceptin binding to cancer cells viewed using the Leica TCS SP2 confocal microscope. Four breast cancer cell lines BT474, ZR-751, MCF-7 and SKBR3 were incubated with FITC labelled Herceptin (green) for 1 hr and the nuclei were counterstained with 1 mM To-Pro3 (blue) as shown. Inset, lower, SKBR-3 cells stained with rabbit anti- cerbB-2 and secondary goat-anti-rabbit antibody labelled with Alexa Fluor 488 (green). The nuclei were counterstained as before (blue). Inset, upper, 3D z-stack of SKBR-3 cells stained with rabbit anti-cerbB-2 and secondary goat-anti-rabbit antibody labelled with Alexa Fluor 488 (green). The SKBR-3 cells stained with the anti-cerbB-2 antibody show the increased accessibility of the HER2 epitope compared with accessibility of the HER2 epitope to Herceptin.

The binding of Herceptin to BT474, ZR-751, MCF-7 and SKBR-3 breast cancer cells was assessed using immunofluorescence and confocal microscopy (Fig. 1). BT474 and ZR-751 showed the least binding to Herceptin while MCF-7 and SKBR-3 cells bound to Herceptin more intensely with SKBR-3 showing the most intense staining amongst these cell lines. When SKBR-3 cells were incubated with an anti-cerbB-2 antibody, as expected, the cells exhibited a much more intense staining pattern due to greater accessibility of the HER2 epitope to the antibody compared with accessibility to Herceptin. All subsequent experiments in which the kinetics of Herceptin binding was assessed were performed with SKBR-3 cells.

**Figure 2:**
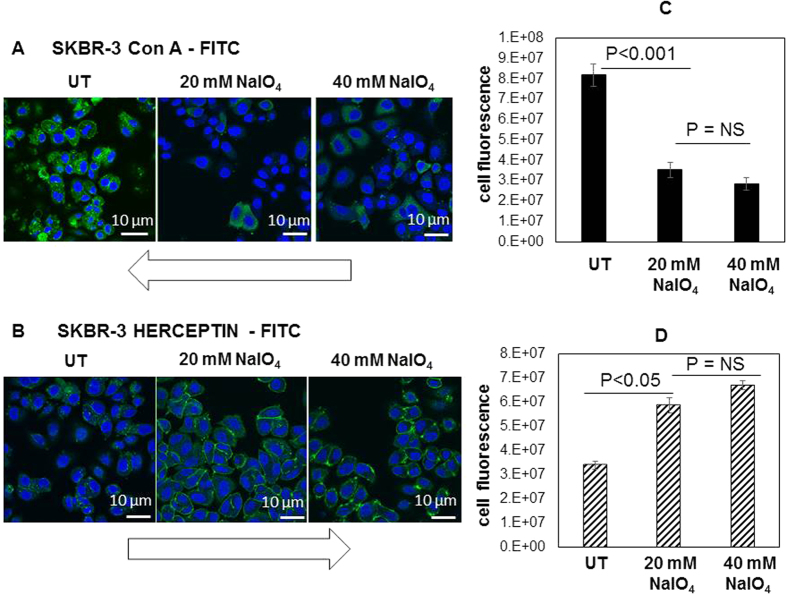
Herceptin and Con A binding to SKBR3 cells untreated and after treatment with 20 mM and 40 mM sodium periodate for 1 h at room temperature. The cells were stained with FITC labelled Con A (green) (**A**) or FITC labelled Herceptin (green). The nuclei were counterstained with 1 mM To-Pro3 (blue). Immunofluorescence images showed a decrease in the binding of Con A in the cytoplasm and increased binding of Herceptin to the cell membrane following treatment of the cells with sodium periodate. Student t-test analysis of total cell fluorescence showed a significant difference between untreated (UT) and treated cells for (**C**) Con A (p < 0.001) binding and (**D**) Herceptin (p < 0.05) binding. Images were analysed using ImageJ (National Institute of Health) and total corrected cell fluorescence was calculated using the integrated fluorescence density (n = 10) after subtraction of the mean average fluorescence of background reading. NS; non significant.

To assess cellular N-linked glycosylation, the lectin Con A from jack bean was employed in conjunction with fluorescence microscopy this confirmed the presence of N-linked glycans located predominately in the perinuclear region of the cytoplasm, consistent with intracellular trafficking of glycoproteins through the endoplasmic reticulum and Golgi apparatus (Fig. 2A). Binding of Herceptin was observed on the cell surface of SKBR-3 in focal regions and at cell junctions (Fig. 2B). Chemical treatment of SKBR-3 cells with sodium periodate (20 and 40 mM) to deglycosylate (oxidise) the glycans resulted in a significant decrease in Con A binding and a significant increase in Herceptin binding (p < 0.05), this change in Herceptin binding led to the experiments below in which the kinetics of Herceptin binding at the cell surface was investigated. The result of experiments undertaken to deglycosylate rHER2 protein are shown in Supplementary Figure 1.

### Herceptin/Con A binding to SKBR3 cells and to rHER2 protein

**Figure 3:**
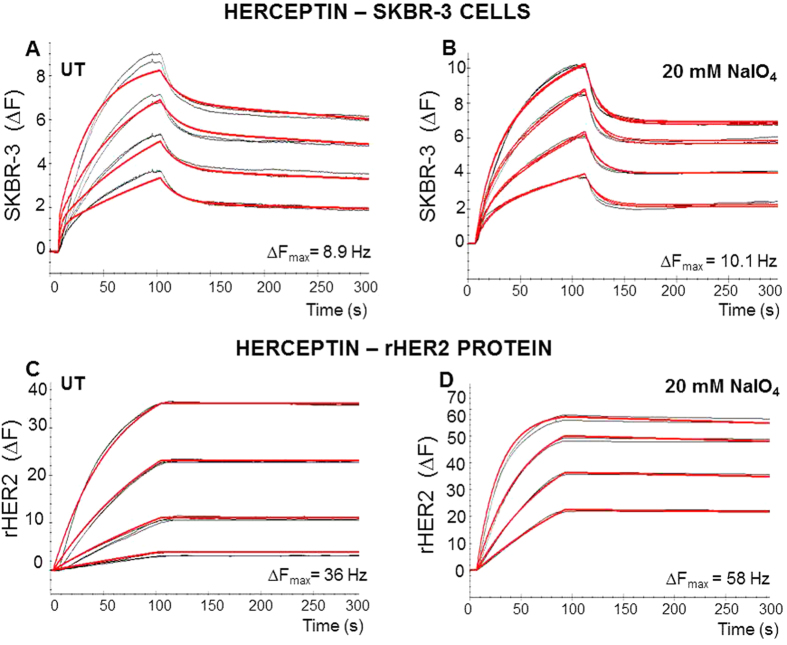
Representative sensograms (black) illustrating real time binding of Herceptin onto SKBR3 cells grown on the QCM sensor chip surface (**A**,**B**) and rHER2 immobilised onto the biosensor surface (**C**,**D**). Cells were treated with 20 mM sodium periodate for 1 hour at room temperature. Herceptin binding was monitored using the Attana Cell 200. A COP-1 chip without cells was also used and the background binding data subtracted from the data for the cell chip. The association (105s) and dissociation (195s) phases of binding were monitored at a flow rate of 20 μl/min and at 22 °C. The red curves represent the theoretical curve fitting using TraceDrawer software, either using 1:1 simple or 1:2 binding models. Herceptin was diluted in PBS running buffer and injected at concentrations of 8.58 nM, 17.61 nM, 34.32 nM and 68.64 nM. Regeneration with 10 mM glycine was performed between each analyte injection.

Similar dissociation constant, affinity values, (K_D_) of 0.56 nM and 0.51 nM were observed for Herceptin binding to rHER2 protein and to SKBR-3 cells respectively (Table 1). An increase in the association rate constant (*k*_a_) for Herceptin was observed when rHER2 and SKBR-3 cells were deglycosylated with 20 mM sodium periodate (untreated cells: 2.46 E^+5^ M^−1^s^−1^; treated cells: 4.31 E^+5^ M^−1^s^−1^; untreated protein: 3.36 E^+5^ M^−1^s^−1^; treated protein: 6.61 E^+5^ M^−1^s^−1^). However, dissociation rate constants (*k*_d_) for Herceptin were also increased when rHER2 and SKBR-3 cells deglycosylated with 20 mM sodium periodate (untreated cells: 1.26 E^−4^ s^−1^; treated cells: 3.42 E^−4^ s^−1^; untreated protein: 1.91 E^−4^ s^−1^; treated protein: 2.41 E^−4^s^−1^). The HER2 binding capacity, B_max_, for Herceptin also increased after deglycosylation of SKBR-3 cells and rHER2 protein (Fig. 3 and Table 1).

**Figure 4:**
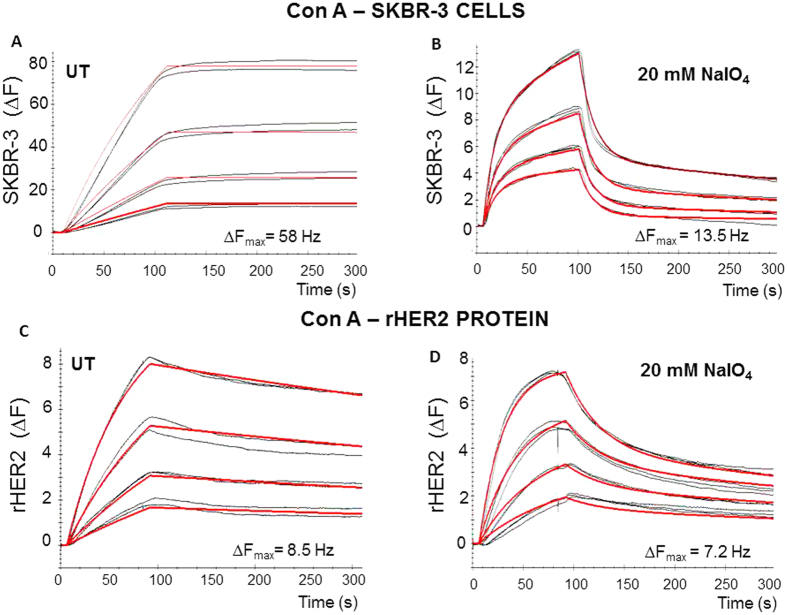
Representative sensorgrams (black) illustrating real time binding of Con A onto SKBR3 cells grown on the QCM sensor chip surface (**A**,**B**) and rHER2 immobilised onto the sensor surface. Cells were treated with 20 mM sodium periodate for 1 hour at room temperature and Con A binding to cell surface was monitored using the Attana Cell 200. LNB-Carboxyl surface activated/deactivated was used as the reference surface. The association (105s) and dissociation (195s) phases of binding were monitored at a flow rate of 20 μl/min and at 22 °C. The red curves represent the theoretical curve fitting using TraceDrawer software, either using 1:1 simple or 1:2 binding models. Con A was diluted in PBS running buffer and injected at concentrations of 2.8 nM, 5.6 nM, 11.1 nM and 22.3 nM. Regeneration was with 10 mM HCl, performed between each analyte injection.

Unsurprisingly, the kinetics of Con A interaction at the SKBR-3 cell surface were significantly reduced when cells were treated with 20 mM sodium periodate. The dissociation rate constant, *k*_d_, increased more than a thousand fold in the treated cells, Fig. 4 and Table 2 (untreated cells: 8.9 E^−6^ s^−1^; treated cells: 1.93 E^−3^ s^−1^) and as a result the affinity, K_D_, was decreased by 90% (untreated cells: 0.362 nM; treated cells: 32.8 nM). The Con A binding capacity, B_max_, was significantly reduced following sodium periodate treatment (125.8 Hz to 14.24 Hz). The *k*_a_ and *k*_d_, association and dissociation rate constants, for Con A binding to sodium periodate treated rHER2 also increased.

### The effect of tunicamycin treatment on sensitivity of cancer cells to DXR and growth factors

**Figure 5:**
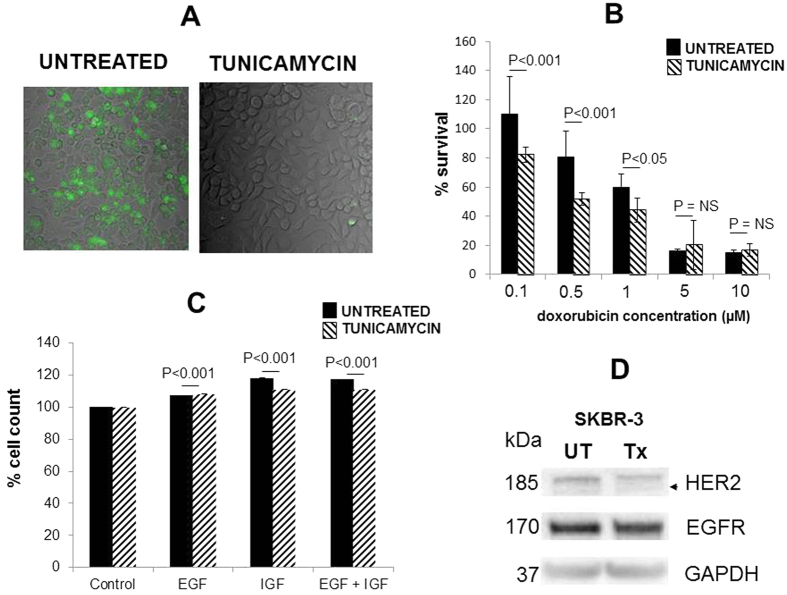
Representative fluorescent images of SKBR3 cells maintained in tunicamycin (1 μg/ml) and stained with WGA conjugated QD at 4 °C for 3 h and SKBR-3 cells maintained in medium without tunicamycin and treated with the same volume of QD alone (**A**). The percentage survival of SKBR-3 cells maintained with/without tunicamycin and following treatment with DXR (1 nM–10 μM) the mean % cell survival was calculated assuming 100% survival for untreated cells (**B**). Cell stimulation as measured by % cell count of SKBR-3 cells maintained with/without tunicamycin and exposed to EGF, IGF alone or in combination (**C**). Western blot analysis of EGFR (HER1) and HER2 for SKBR-3 cells pre and post treatment with tunicamycin a faint band which may represent deglycoyslated HER2 in the tunicamycin treated cells is shown by the arrow (**D**). UT: Untreated, Tx: tunicamycin treated GAPDH levels were assessed as a gel loading control.

SKBR-3 cells were maintained in tunicamycin and the lectin WGA, conjugated to QDs, was used to monitor the reduction in glycosylation (Fig. 5A). The cells were cultured in tunicamycin (100 nM–1 μM) over several passages (Fig. 5B) and the metabolic activity was assessed using an MTT assay When cells were exposed to dual treatment with DXR (100 nM, 0.5 μM, and 1 μM) and tunicamycin a significant decrease in the metabolic activity was observed (student t-test, unpaired, P < 0.001). No significant difference in the metabolic activity was observed when the cells were cultured with DXR at 5 and 10 μM and tunicamycin. The IC_50_ value for SKBR-3 cells treated with tunicamycin was determined: treated cells: 0.57 μM DXR: untreated: 0.91 μM DXR, Supplementary Figure 2. To further investigate the effect of glycosylation on cancer cell responsiveness to extrinsic factors SKBR-3 cells were maintained in tunicamycin and exposed to IGF-1 and EGF (Fig. 5C) and the cellular activity monitored using the MTT assay. Cells maintained in tunicamycin were significantly less responsive to IGF-1 alone (decreased responsiveness by 7%, paired t-test, p < 0.001) or in combination with EGF (decreased responsiveness by 6.3%, paired t-test, p < 0.001). Somewhat surprisingly, this observation was not made when the cells were maintained in tunicamycin and treated with EGF alone (Fig. 5C) despite apparent equal levels of EGFR in the tunicamycin treated cells (Fig. 5D). From these observations it can be surmised that inhibition of cellular glycosylation by tunicamycin treatment reduces cellular responsiveness to growth factors. Maintaining cells in tunicamycin reduced the N-linked glycosylation and resulted in a reduction in sensitivity to IGF-1 alone and IGF-1 in combination with EGF.

## Discussion

The influence of N-linked glycosylation on cancer cell responsiveness to common cancer treatment modalities were explored in this study. In particular, the interaction between the biological (monoclonal antibody) treatment Herceptin with HER2 receptors on cancer cell surfaces and the sensitivity of cancer cells to the chemotherapeutic agent DXR was considered alongside the effect of glycosylation on cancer cell sensitivity to the growth factors EGF and IGF-1.

The lectins Con A and WGA were both utilised as tools for monitoring the N-linked glycosylation status of the rHER2 and the breast cancer cell lines. Con A binds mannose residues whilst WGA recognises both GlcNAc and sialic acid residues, as these glycans are present in N-linked oligosaccharides each lectin will provide information on the N-linked glycosylation status of proteins and cells. At the outset, a range of breast cancer cell lines were incubated with FITC-labelled Herceptin, the relatively weak intensity of staining highlighted the inaccessibility of the Herceptin binding site on HER2 in contrast to the binding site for conventional anti- cerbB-2 antibodies (such as utilised in this study). However, when SKBR3 cells were treated with sodium periodate the binding of Herceptin increased as a result of improved accessibility of the HER2 epitope. Changes in the kinetics of Herceptin binding were also observed after deglycosylation of both SKBR3 cells and the rHER2 protein. The dissociation constant, affinity (K_D_) of Herceptin to immobilised rHER2 protein (0.56 nM) and to SKBR-3 cells (0.51 nM), was consistent with previous reports obtained using surface plasmon resonance (SPR) technology (0.5 nM K_D_)^30^. Alterations in the cellular glycan repertoire following deglycosylation resulted in a significant increase in the B_max_ reading. This data suggests that removal of glycans renders the Herceptin binding sites on HER2 more accessible, perhaps due to a lack of stearic hindrance from glycans present either on HER2 itself and/or, in the case of the study with the cell biosensor chip, on neighbouring macromolecules. Removal of the N-glycan residues from the HER2 receptor might lead to a decrease in stability of the Herceptin-HER2 complex and might explain the notable increase in the dissociation rate constant. A recent report comparing the kinetics of binding of Herceptin to SKBR-3 cells with a Herceptin-resistant and sensitive phenotype (analysed using surface plasmon resonance imaging) described an increase in both the *k*_a_ and *k*_d_ in the Herceptin-resistant cell populations, these changes in *k*_a_ and *k*_d_ are consistent with our findings^31^.

A significant increase in the sensitivity of SBKR-3 cells to DXR and a decrease in sensitivity to IGF-1 alone and in combination with EGF were observed on treatment of the cells with tunicamycin. These findings provide evidence of the importance of glycosylation and how it might affect the efficacy of therapeutics in breast cancer and also highlights the potential for approaches aimed at abrogating glycosylation as a means of rendering cancer cells more susceptible to cancer treatment modalities. Fluorescence images of cells stained with WGA-labelled QDs following treatment with tunicamycin showed the effectiveness of this approach for decreasing cellular glycosylation. The use of lectins conjugated to QDs to image glycosylation levels in cells has been reported elsewhere^32^ and further work has shown their potential for exploring the relationship between glycosylation changes and metastatic potential in cancer^33^. The work performed here builds on a growing body of evidence suggesting that lectins conjugated to QDs may have widespread application as tools for cancer diagnostics. As the understanding of glycoproteomic changes in cancer biology grows future applications may involve QDs with more specific glycosylation targets. MUC1 for example, has been identified as a possible nano-particle imaging target, with both prognostic value as well as therapeutic application^34^.

The human HER2 receptor has been predicted to be glycosylated with seven N-linked glycans (Supplementary Figure 1). N-linked glycosylation of HER2 is required for successful translocation of HER2 to the cell surface and acquisition of function^35^. The importance of N-linked glycosylation of HER2 for downstream signalling via the MAPK pathway has also been shown previously^36^. When SKBR-3 cancer cells were treated with sodium periodate a significant increase in the binding of Herceptin was observed. QCM biosensor analysis showed that this was due to an increased rate of Herceptin binding and an increase in the B_max_, implying improved accessibility of the Herceptin binding domain on HER2 following protein deglycosylation. This increased accessibility is consistent with a de-masking of the Herceptin binding epitope of HER2 on cancer cell surfaces. Whilst the observed increase in B_max_ may be due to deglycosylation of HER2 itself, it is also possible that deglycosylation of other neighbouring cell surface glycoproteins (for example, MUC4, HER1, HER3, HER4 and IGFR) might underlie the reason(s) for the increased accessibility of the Herceptin binding domain on HER2. When rHER2 was immobilised onto the QCM biosensor chip surface a similar observation was made further supporting the hypothesis that glycosylation affects the accessibility of the HER2 protein binding domain to Herceptin. The data obtained in this study concur with a recent report in which SKBR-3 and MCF-7 cells were treated with tunicamycin and for which an increased responsiveness of the cells to Herceptin was observed, correlating with increased cell cycle arrest and cellular apoptosis^37^.

**Figure 6:**
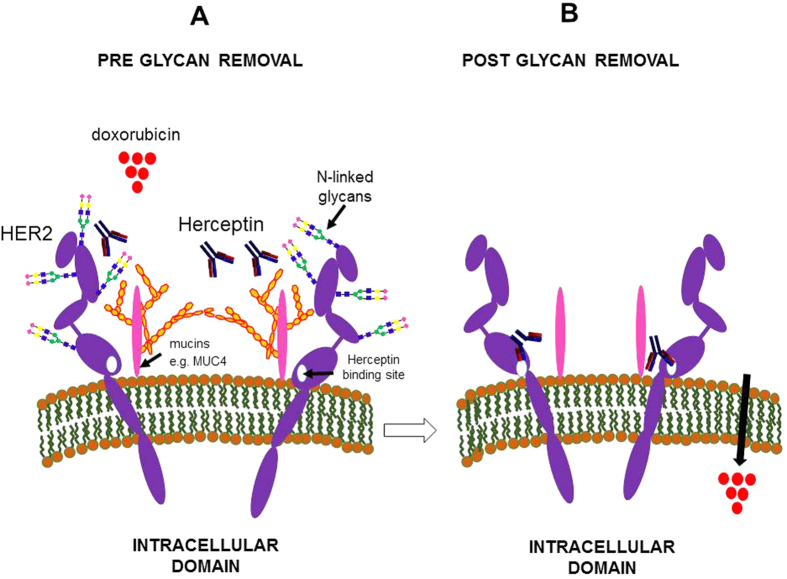
Schematic diagram showing the extracellular domain of the SKBR-3 cells, the HER2 protein prior to (**A**) and after (**B**) glycan removal. Masking of Herceptin binding to the juxtamembrane domain of HER2 receptor and DXR access to transporter proteins (eg: RL1P76) by large membrane glycoproteins such as mucins (MUC4) or N-linked glycans on the receptor.

Tunicamycin treatment of SKBR-3 cells resulted in a decreased response to the growth factor IGF-1 alone and in combination with EGF. This may be due to decreased IGFR-R1 function following deglycosylation as reported elsewhere^38^ or due to down-regulation in IGF-R1 receptor levels as observed when melanoma cells were maintained in tunicamycin^39^. A further possible mechanism for the increased sensitivity of SKBR-3 cells to DXR is that with reduced glycosylation the overall cellular glycocalyx offers less of a barrier to chemotherapeutic agents as maintenance of SKBR-3 cells in tunicamycin resulted in cancer cells that were more sensitive to treatment with DXR at a range of concentrations. No significant difference in the metabolic activity of the cells was observed at higher concentrations of DXR (5 and 10 μM) and this may be due to the cytotoxicity of the DXR at these concentrations. The findings of this study provide additional evidence for the potential use of glycosylation inhibitors as a supplement to current chemotherapeutic regimens. Reports attest to the increased susceptibility of cancer cells to chemotherapeutic agents when treated with such inhibitors, for example, the multi-drug resistant ovarian cancer cell line UWOV2 became responsive to vincristine, cisplatin and DXR on tunicamycin treatment^40^. Induction of cell cycle arrest and increased apoptosis has been reported following Herceptin treatment of breast cancer cells grown *in vitro* and *in vivo* and treated with tunicamycin^30^. Similarly, increased sensitivity of cancer cells to cisplatin both *in vitro* and in a cisplatin resistant (C3H/HE) mouse model *in vivo* has been reported following tunicamycin treatment^41^. The responses may be tumour/cell type specific, however, as it has been noted that tunicamycin treatment was associated with an increase in resistance to chemotherapy agents in hepatocellular carcinoma cells^42^. Since tunicamycin has a broad effect on all N-linked glycoproteins, tunicamycin-induced inhibition of N-glycosylation may produce systemic side effects if used as a treatment modality although optimal tunicamycin doses which do not induce cell toxicity have been achieved using MCF-7/HER2 xenograft models^30^. Similarly, inhibition of N-linked glycosylation of the P-glycoprotein has been achieved without altering its function as a drug efflux pump^43^. Whole patient treatment with swainsonine to inhibit alpha mannosidase has shown the potential tolerability of this approach^44^ supporting the role for glycosylation inhibitors as combination therapies for cancer^45^. The reduction in N-linked glycosylation of SKBR-3 cells also resulted in a reduced proliferative response to growth factors and increased sensitivity to DXR. The findings highlight the importance of the glycocaylx in the accessibility of the HER2 epitope to Herceptin as glycosylation occurs on many proteins and lipids this may affect many aspects of cancer cell biology and drug responsiveness (Fig. 6).

In conclusion, this study highlights the importance of cellular glycosylation on the binding of the drug Herceptin to the surface of cancer cells, the responsiveness of cancer cells to the chemotherapeutic agent DXR and sensitivity to growth factors. The findings illustrate the importance of glycosylation in modulating the responsiveness of cancer cells to treatments. The study also highlights a potential role for glycosylation inhibitors as (combination) treatments for breast cancer.

## Materials and Methods

Chemicals were obtained from Sigma-Aldrich, Poole, Dorset, UK, unless otherwise stated. All cell lines were recently obtained from the ATCC and are part of a collection of breast cancer cells held at the University of Westminster and University College London. The cells have been confirmed mycoplasma free. SKBR-3, MCF-7, ZR75–1 cells were subjected to short tandem repeat (STR) typing performed in-house using the Genome Lab Human STR Primer Set (Beckman Coulter, High Wycombe, UK). DNA samples were prepared using the high-pure PCR template preparation kit (Roche, Welwyn Garden City, UK). Fragment analysis was performed on a Beckman Coulter CEQ8800 Genetic Analyser and the STR genotype profile of each cell line was analysed using an online tool provided by the DMSZ collection of cell cultures https://www.dsmz.de/.

### Cell culture

Four human breast cancer cell lines were used: SKBR3, MCF-7, BT474 and ZR-175. SKBR-3 is a human epithelial cell line that overexpresses the HER2 receptor^29^ and has been widely used for HER2 expression studies. BT474 is also a HER2 overexpressing cell line. Both MCF-7 and ZR-175 are known to be HER2 negative. SKBR-3 cells were cultured in McCoy’s 5 A medium with 2 mM L-glutamine (Lonza, Slough, UK) supplemented with 10% (v/v) foetal calf serum (FCS; Biosera, Ringmer, UK). The remaining cell lines were grown in DMEM (Lonza, Slough, UK) supplemented with 10% (v/v) FCS. All cells were incubated at 37 °C in humidified atmosphere in a 5% v/v CO_2_ incubator. The cells were passaged at the split-ratio recommended by the ATCC and were seeded into 6-well plates using standard cell culture techniques. All the experiments were performed within a total of ten passages.

### Immuno-fluorescent staining and confocal microscopy

The accessibility of the HER2 receptor to Herceptin was investigated in BT474, ZR-751, MCF-7 and SKBR-3 cells using fluorescein isothiocyanate (FITC) labelled Herceptin (Roche, Welwyn Garden City, UK, 160218-150 mg) prepared using a FITC labelling kit according to the manufacturer’s recommendations (Piercenet, Illinois, USA) this included a desalting step on a PD-10 column (GE Healthcare, Amersham, UK). The cells were grown to 80% confluence on the surface of 6 well plates, washed with phosphate buffered saline (3x, PBS, pH 7.6), fixed in 3.7% formaldehyde/PBS, again washed with PBS and blocked with 5% w/v bovine serum albumin (BSA) for 30 min. Fixation of cells in formaldehyde renders the HER2 epitope inaccessible to Herceptin. A range of treatment methods were tested for effective Herceptin staining; treatment of cells with porcine trypsin (1 mg/ml) for 20 min at 37 °C was established as the most effective antigen-retrieval approach. Cells were incubated with FITC-labelled Herceptin, 10 μg/ml, for 1 hour in the dark. In addition, SKBR-3 cells were stained with anti-cerbB-2 antibody, 0.614 μg/ml, for 1 hour at room temperature (Abcam, Cambridge, UK) and secondary anti-rabbit IgG, 4 μg/ml for 1 hour at room temperature (Alexa Fluor 488, Abcam, Cambridge, UK). Lectin staining was performed in the absence of trypsinisation with rhodamine or FITC labelled Con A or WGA (10 μg/ml) (Vector Labs, Peterborough, UK) for 1 h. Cells were counterstained with the nuclear stain To-Pro-3 (1 mM) for 30 min after pre-treatment with RNase A (10 μg/ml) for 20 min. Images were acquired by sequential scanning using a Leica TCS SP2 confocal system (Leica Microsystems, Milton Keynes, UK) and analysed using ImageJ (National Institute of Health, Maryland, U.S.A.). Z-stacks were taken with each slice thickness 1 μm. The total corrected cell fluorescence was calculated using the integrated fluorescence density (n = 10) following subtraction of the mean background fluorescence reading.

### Deglycosylation

The antibiotic tunicamycin was used to inhibit cellular glycosylation at a final concentration of 1 μg/ml; cells were maintained in the treatment medium ≥ 7 passages. The effectiveness of this step was assessed by fluorescent lectin cytochemistry using WGA conjugated to quantum dots (QD) prepared, in brief, using cadmium chloride and sodium tellurite to form the core, with a mercaptosuccinic acid coating, in sodium borate/citrate buffer, pH 7.2 and sodium borohydride as a reductant^46^,^47^ the QDs were washed, suspended in PBS and refrigerated at 2 °C until use. Where chemical deglycosylation was employed to oxidise the glycans, cells were treated with 20–60 mM sodium periodate, pH 5.5, for 1 h^48^. The effectiveness of this step was assessed by fluorescent lectin cytochemistry with Con A as described above.

### Biosensor experiments

QCM biosensor experiments were performed using an Attana Cell 200 instrument using consumables and software (Attana, Stockholm, Sweden) unless otherwise stated. 40,000 SKBR-3 cells were seeded onto a cell culture compatible polystyrene coated sensor surface (COP-1) allowed to settle and maintained under standard cell culture conditions for 18 h, the cell chips were washed in PBS (3X) and fixed in fresh 3.7% v/v formaldehyde/PBS for 20 min at room temperature. To evaluate the cell coverage, the nuclei were stained with To-Pro3 as described above and visualised using confocal microscopy. A COP-1 surface processed the same way as the cell chip (without cells) was used as a negative control.

rHER2 protein (Sino Biologics Inc., Beijing, China), 2 μg, was reduced using 10 mM DTT and immobilised onto an LNB-Carboxyl sensor surface by amine coupling using EDC and sNHS according to the manufacturer’s instructions (Attana, Stockholm, Sweden). Remaining active groups were deactivated using 1 M ethanolamine for 300 sec. A LNB-Carboxyl surface activated and deactivated as above was used as the reference surface. Two fold serial dilutions of Herceptin (8.5 nM–17.1 nM) and Con A (2.7 nM–44 nM) were used to study the kinetics of binding to immobilised HER2 and to the cell surface at a flow rate of 20 μl/min at 22 °C. Between each Herceptin dilution the surface was regenerated using 10 mM glycine pH 2.2 and between each Con A dilution the surface was regenerated using 10 mM HCl; the same procedure was followed to regenerate the cell chip surfaces.

The changes in the frequency of the sensor surface resonance (ΔF) during the binding experiments was recorded using the Attestar software and the data were analysed using the Evaluation (Attana, Stockholm, Sweden) and TraceDrawer software (Ridgeview Instruments AB, Stockholm, Sweden). To obtain values for the specific binding response, the background binding to the reference surfaces were subtracted from the experimental surfaces. 1:1 or 1:2 binding models were used to calculate the kinetic parameters including the rate constants (*k*_a_, *k*_d_), dissociation constant (K_D_) and the maximum binding capacity (B_max_). All biosensor experiments were repeated using a second and third set of sensor surfaces prepared using the same procedure.

### Cell Inhibition/Stimulation and MTT assays

Cells were seeded onto 24-well plates in media (as above) at 5 × 10^4^ cells per well for inhibition assays or 2.5 × 10^4^ cells per well for growth assays, and allowed to settle for 24 hours. The cells were semi-synchronized to the same phase of the cell cycle by replacing the medium with McCoy’s 5 A with 2 mM L-glutamine and incubating for a further 24 hours. The medium was supplemented with 0.5% v/v dextran-coated charcoal stripped serum and EGF and IGF-1 were added alone or in combination to a final concentration of 1 ng/ml, or 50 ng/ml respectively. The metabolic activity of the cells was assessed using an MTT (3-(4,5-dimethylthiazol-2-yl)-2,5-diphenyltetrazolium bromide) assay in a 96-well plate format. MTT was used at a final concentration of 0.5 mg/ml per well followed by incubation at 37 °C for 2 hours (n = 8). The experiments were repeated under identical conditions on separate days. The insoluble tetrazolium salt was reduced to the soluble formazan dye by addition of 600 μl dimethyl sulphoxide and further incubation of the plate for 10 min at room temperature. The optical density was read using a colorimetric plate reader (BioTek Instruments, USA) at 570 nm absorbance. The IC_50_ values of DXR for both treated and untreated cells were calculated using the GraphPad Prism Software.

### Protein extraction, SDS-PAGE and Western blotting

Cells were harvested at 80% confluence into radio-immunoprecipitation assay (RIPA) buffer containing protease inhibitors (Roche Diagnostics, Germany). The cellular debris was pelleted by centrifugation (20,000 ×*g* for 5 min) and protein concentration quantified using the Lowry protein assay. Equal quantities of protein (20 μg) heat-denatured in the presence of 0.03% v/v β-mercaptoethanol and 1X NuPAGE LDS Sample Buffer (Fisher Scientific, Loughborough, UK) in a total of 30 μl was loaded onto a NuPAGE Novex 4–12% gradient Bis-Tris gel (Fisher Scientific, Loughborough, UK) and separated by electrophoresis. Western blotting was performed by transfer of the separated proteins by electroblot to a (0.22 μm) PVDF (polyvinylidene fluoride) membrane (BioRad, Hemel Hempstead, UK), the membrane was blocked using 2.5% w/v BSA in PBS − 0.05% v/v Tween-20 for 30 min.

Protein expression levels were confirmed using primary anti-HER2/Neu mouse monoclonal antibody (Santa Cruz; Dallas, Texas, US sc-08, 1:500 dilution in 1% w/v BSA-Tween) and anti-EGFR mouse monoclonal antibody (Santa Cruz; Dallas, Texas, US, sc-374607 1:1000 dilution in 1% w/v BSA-Tween) followed by goat anti-mouse horseradish peroxidase conjugated antibody (Santa Cruz Dallas, Texas, US sc-2005; 1:2500 dilution in 1% v/v BSA-Tween) at room temperature. The HRP reaction was detected using Clarity^TM^ Western ECL substrate and visualised using ChemiDoc XRS with Image Lab software (Bio-Rad, Hemel Hempstead, UK).

### Statistical Analysis

Microsoft Excel 2013 was used to generate graphical data and descriptive statistics. IBM SPSS Statistics version 21 was used for all other statistical analysis.

## Additional Information

**How to cite this article:** Peiris, D. *et al*. Cellular glycosylation affects Herceptin binding and sensitivity of breast cancer cells to doxorubicin and growth factors. *Sci. Rep.* **7**, 43006; doi: 10.1038/srep43006 (2017).

**Publisher's note:** Springer Nature remains neutral with regard to jurisdictional claims in published maps and institutional affiliations.

## Supplementary Material

Supplementary Figures

## Figures and Tables

**Table 1:** Kinetic parameters derived from sensograms of Herceptin binding to either SKBR3 cell surfaces or rHER2 protein using TraceDrawer software, either using 1:1 simple or 1:2 binding models.

SKBR-3 CELLS				
	*k*_a_ (M^−1^s^−1^)	*k*_d_ (s^−1^)	KD (nM)	Bmax
Untreated	2.46 E^+5^ (±9.41)	1.26E^−4^ (±2.32 E^−5^)	0.512 (±0.09)	6.83 (±5.82 E^−5^)
20 mM sodium periodate	4.31 E^+5^ (±7.0)	3.42 E^−4^ (±1.9 E^−5^)	0.795 (±0.04)	7.35 (±1.37 E^−3^)
rHER2 PROTEIN				
Untreated	3.36 E^+5^ (±2 E^+1^)	1.91 E^−4^ (±4.91 E^−5^)	0.56 (±0.146)	42 (±0.003)
20 mM sodium periodate	6.61 E^+5^ (±1.12 E^+1^)	2.14 E^−4^ (±1.4 E^−7^)	0.323 (±0.2)	62.4 (±0.014)

Mean average values ± SD, derived from two sensor chips for each condition and four analyte concentrations.

**Table 2:** Kinetic parameters derived from sensograms of Con A binding to either SKBR3 cell surfaces or rHER2 protein using TraceDrawer software, either using 1:1 simple or 1:2 binding models.

SKBR-3 CELLS				
	*k*_a_ (M^−1^s^−1^)	*k*_d_ (s^−1^)	KD (nM)	Bmax
Untreated	2.46 E^+4^ (±11.92)	8.9 E^−6^ (±6.23 E^−5^)	0.362 (±0.07)	125.8 (±0.007)
20 mM sodium periodate	5.8 E^+4^ (±1.49 E^+3^)	1.93 E^−3^ (±1.08 E^−5^)	32.8 (±1.02)	14.24 (±0.022)
rHER2 PROTEIN				
Untreated	1.75 E^+5^ (±3.62 E^+1^)	8.76 E^−4^ (±1.48 E^−6^)	5.02 (±0.03)	11.17 (±0.08)
20 mM sodium periodate	1.51 E^+5^ (±2.85 E^+2^)	1.89 E^−4^ (±5.12 E^−7^)	1.25 (±0.004)	5.03 (±0.05)

Mean average values ± SD, derived from two sensor chips for each condition and four analyte concentrations.
