# Supplementary material for: Cellular glycosylation affects Herceptin binding and sensitivity of breast cancer cells to doxorubicin and growth factors
Source: Sci Rep. 2017 Feb 22;7:43006. doi: 10.1038/srep43006 (PMC5320443; doi:10.1038/srep43006)
Supplement: Supplementary Figures [file srep43006-s1.doc]

**SUPPLEMENTARY INFORMATION**

**TITLE:**

**Cellular glycosylation affects Herceptin binding and sensitivity of breast cancer cells to doxorubicin and growth factors.**

Diluka Peiris^a^, Alexander F. Spector^b^, Hannah Lomax-Browne^c^, Tayebeh Azimi^c^, Bala Ramesh^b^, Marilena Loizidou^b^, Hazel Welch^b^, Miriam V. Dwek^c^

^a^Attana AB, Bjornnasvagen 21, SE-11419, Stockholm, Sweden.

^b^Division of Surgery and Interventional Science, UCL Medical School Royal Free Campus, Rowland Hill Street, London, NW3 2PF, UK.

^c^Department of Biomedical Sciences, Faculty of Science and Technology, University of Westminster, 115 New Cavendish St, W1W 6UW, UK.

**SUPPLEMENTARY FIGURE 1**

SDS-PAGE analysis following the deglycosylation of rHER2. Lane 1: molecular weight markers, lane 2: rHER2, lane 3: rHER2 treated with 20 mM sodium periodate, lane 4: rHER2 treated with 500 mU PNGase F , lane 5: 500 mU PNGase F alone, lane 6: rHER2 treated with 40 mU O--glycosidase. Digestion with PNGase F resulted in a change in the migration of the rHER2 protein, as a result of removal of N-linked glycans. 1 µg of treated or untreated protein was loaded into each well of the gel prior to SDS-PAGE separation. The gel was stained with Coomassie brilliant blue (A). Western blot analysis of rHER2 pre and post sodium periodate and PNGaseF treatment as indicated. The blot was probed using biotinylated Herceptin, incubated with streptavidin-peroxidase and visualized using diaminobenzidine/H2O2 (B). Analysis of potential N-linked and O-linked glycosylation sites of HER2 protein predicted using the NetNGlyc (http://www.cbs.dtu.dk/services/NetNGlyc/) and the NetOGlyc (http://www.cbs.dtu.dk/services/NetOGlyc/) tools. Asparagine residues predicted to be N-glycosylated are highlighted in red and Asn-Ser/Thr sequons are highlighted in blue (C).

**
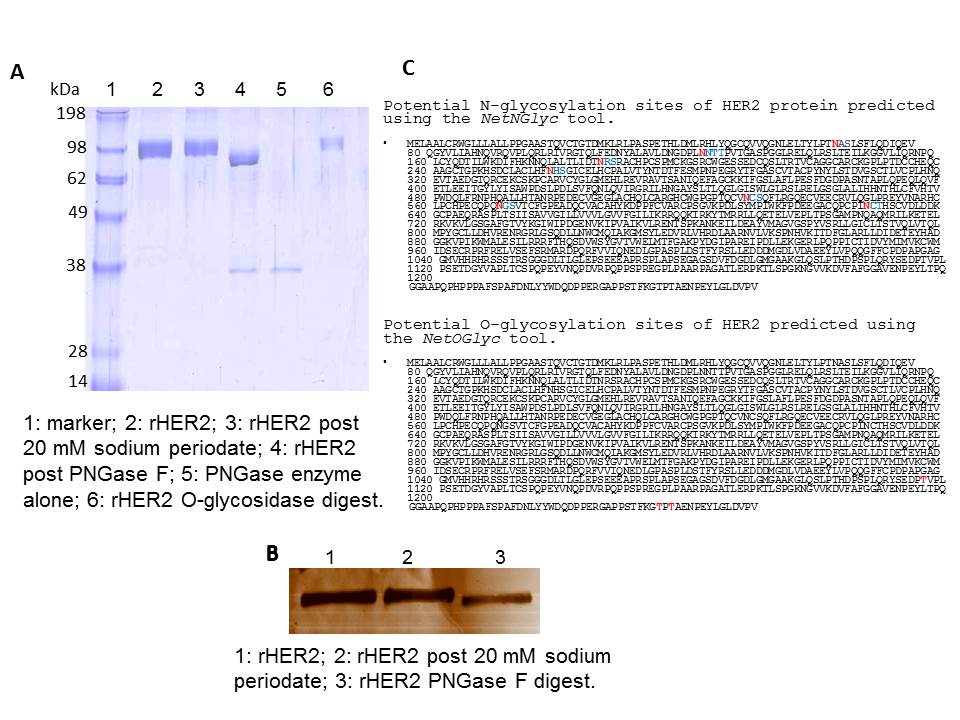
**

**SUPPLEMENTARY FIGURE 2.**

IC50 values for tunicamycin treated and untreated SKBR3 cells, calculated from the data from the MTT assay.

**
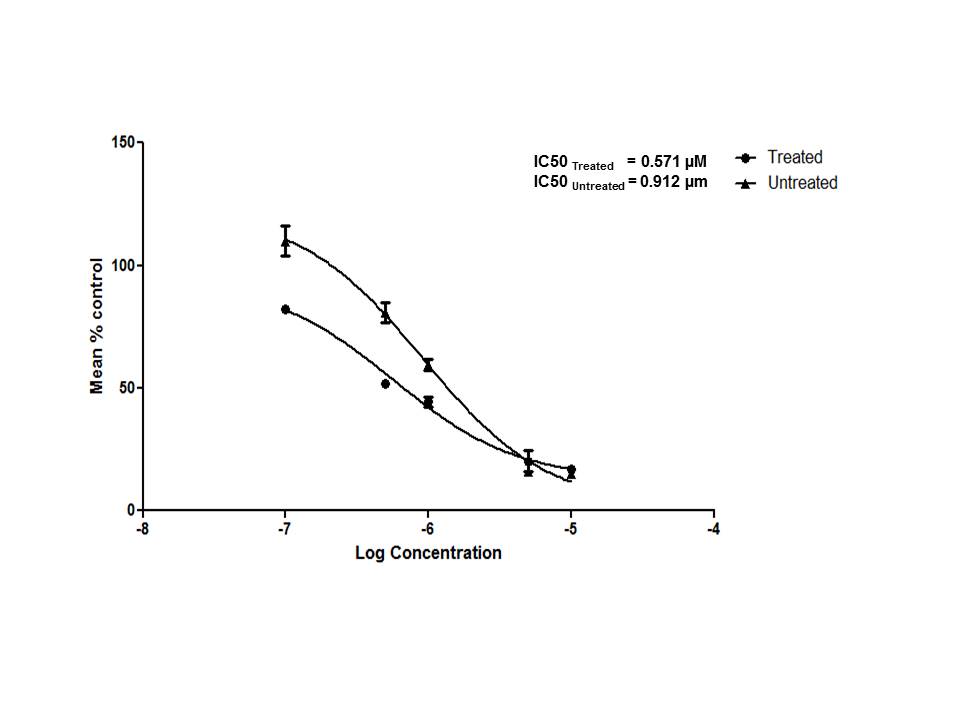
**
